# Supplementary material for: Formation of a hard surface layer during drying of a heated porous media
Source: PLoS One. 2020 Feb 27;15(2):e0229723. doi: 10.1371/journal.pone.0229723 (PMC7046278; doi:10.1371/journal.pone.0229723)
Supplement: S1 File — (DOCX) [file pone.0229723.s001.docx]

**Formation of a hard surface layer during drying of a heated porous media**

*Navneet Kumar, Jaywant H Arakeri, Musuvathi S Bobji*

*Department of Mechanical Engineering*

*Indian Institute of Science, Bangalore – 560012, India*

*^#^Correspondence to Navneet Kumar:* [*navneetkumar@iisc.ac.in*](mailto:navneetkumar@iisc.ac.in) , [navneet01011987@gmail.com](mailto:navneet01011987@gmail.com)

**Supplementary Figures:**


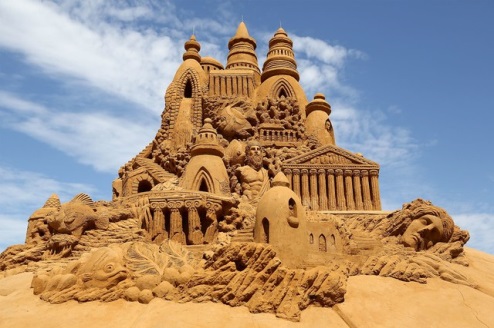

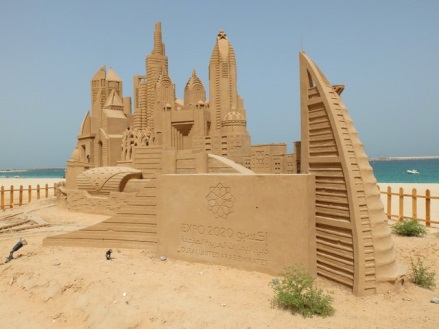

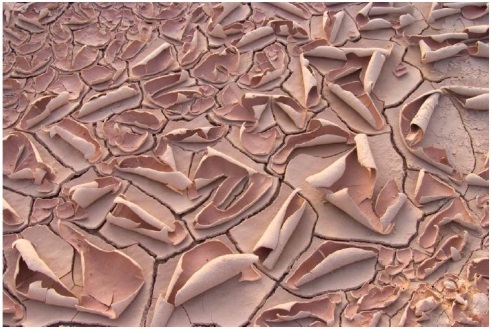


(a)

(c)

(b)

Figure S1 Photographs of sandcastles and mud-peels. Tiny amount of water in sand helps its particles bind together so as to withstand against gravity. Sharp structures are seen in (a) and (b). Leaching followed by the formation of mud-peels is seen nearly 2 mm thick and are fragile. (c) is taken from Style et al. (2011).


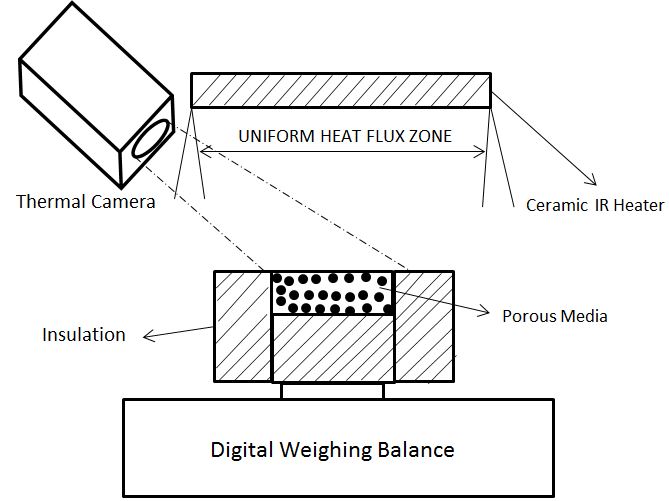


Figure S2 Schematic of the experiment setup. A digital precision weighing balance is used to monitor mass loss, Ceramic IR heater heats the evaporating surface from above. A thermal camera, fixed at an angle, measures the surface temperature of the top surface of the porous medium.


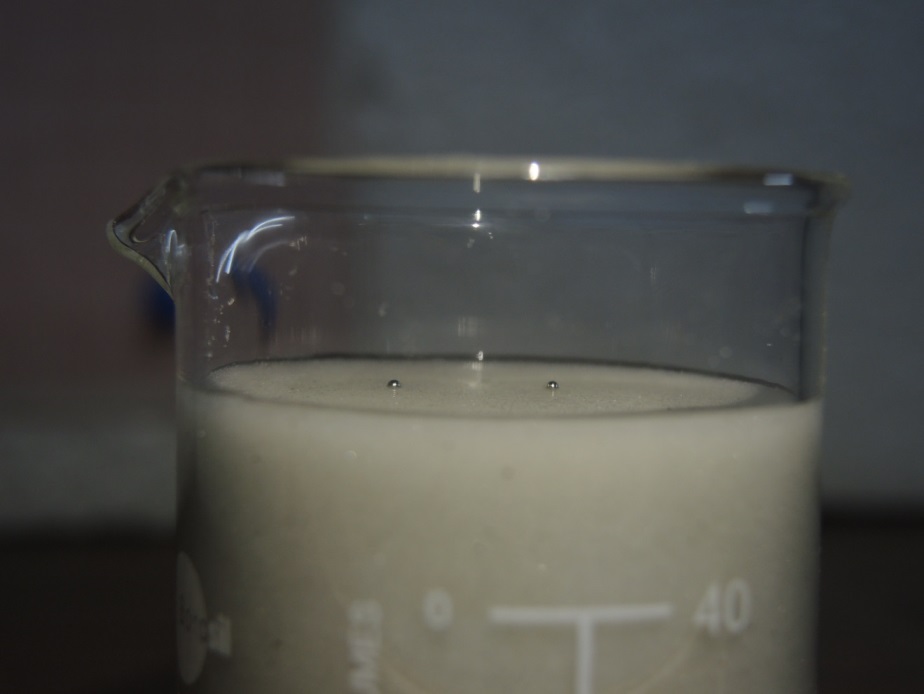


Figure S3 A close-up view of the top surface of the nearly saturated porous medium. Two stainless steel (SS) balls are seen embedded on the top surface. These balls are dropped vertically from ~10 cm away. Since the porous medium is nearly saturated, the SS balls do not bounce back rather they made an indent on the surface on impact. This behaviour of the porous medium is similar to that of sandcastles although the latter are partially saturated.


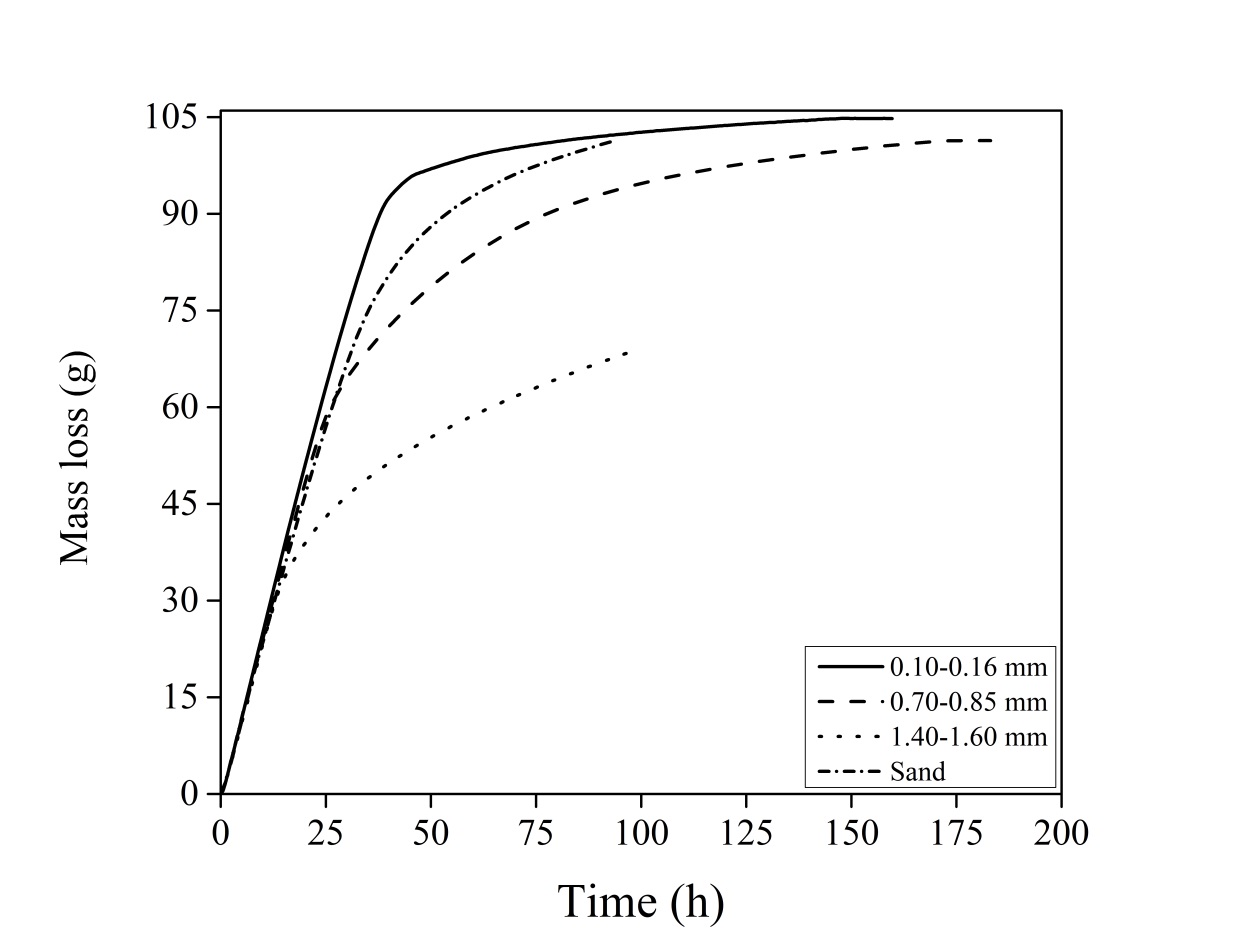


Figure S4 Variation of the evaporated mass versus time for different cases. Heat flux received by the top surface in all the cases was ~1000 W/m^2^.


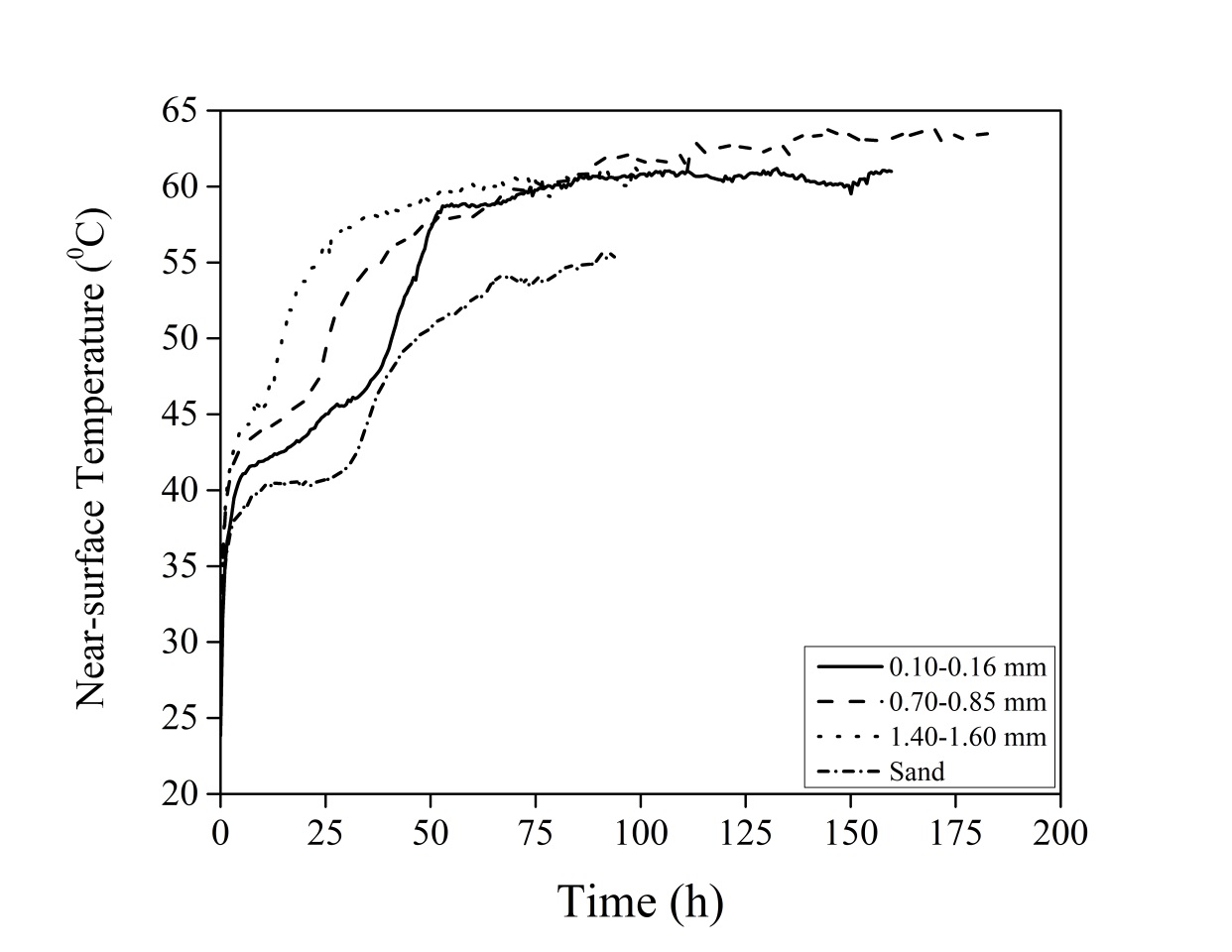


Figure S5 Variation of the near-surface temperature versus time for the different cases. Heat flux received by the top surface in all the cases was ~1000 W/m^2^.


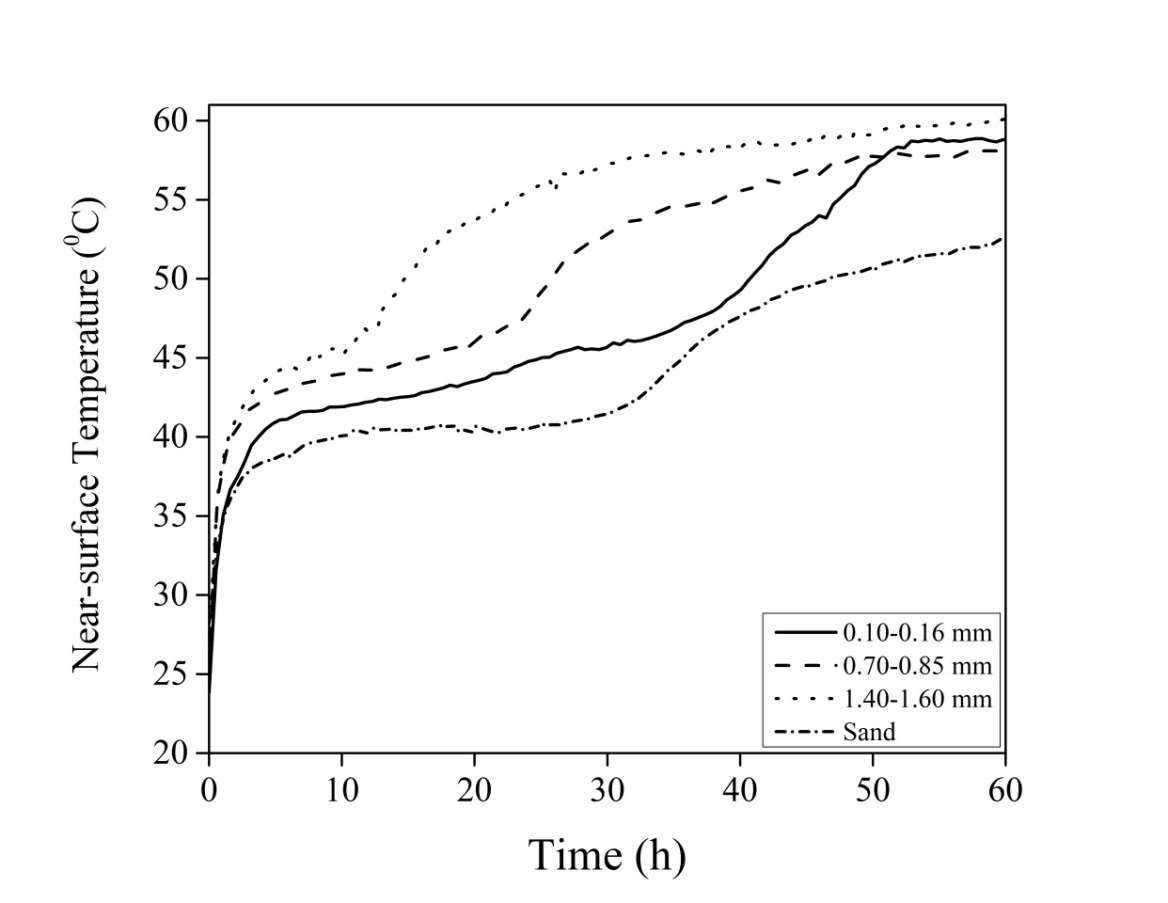


Figure S6 Same as Figure S5 but here the time scale has been limited to 60 hours. This helps us in distinguishing accurately the evaporation rates among different cases.


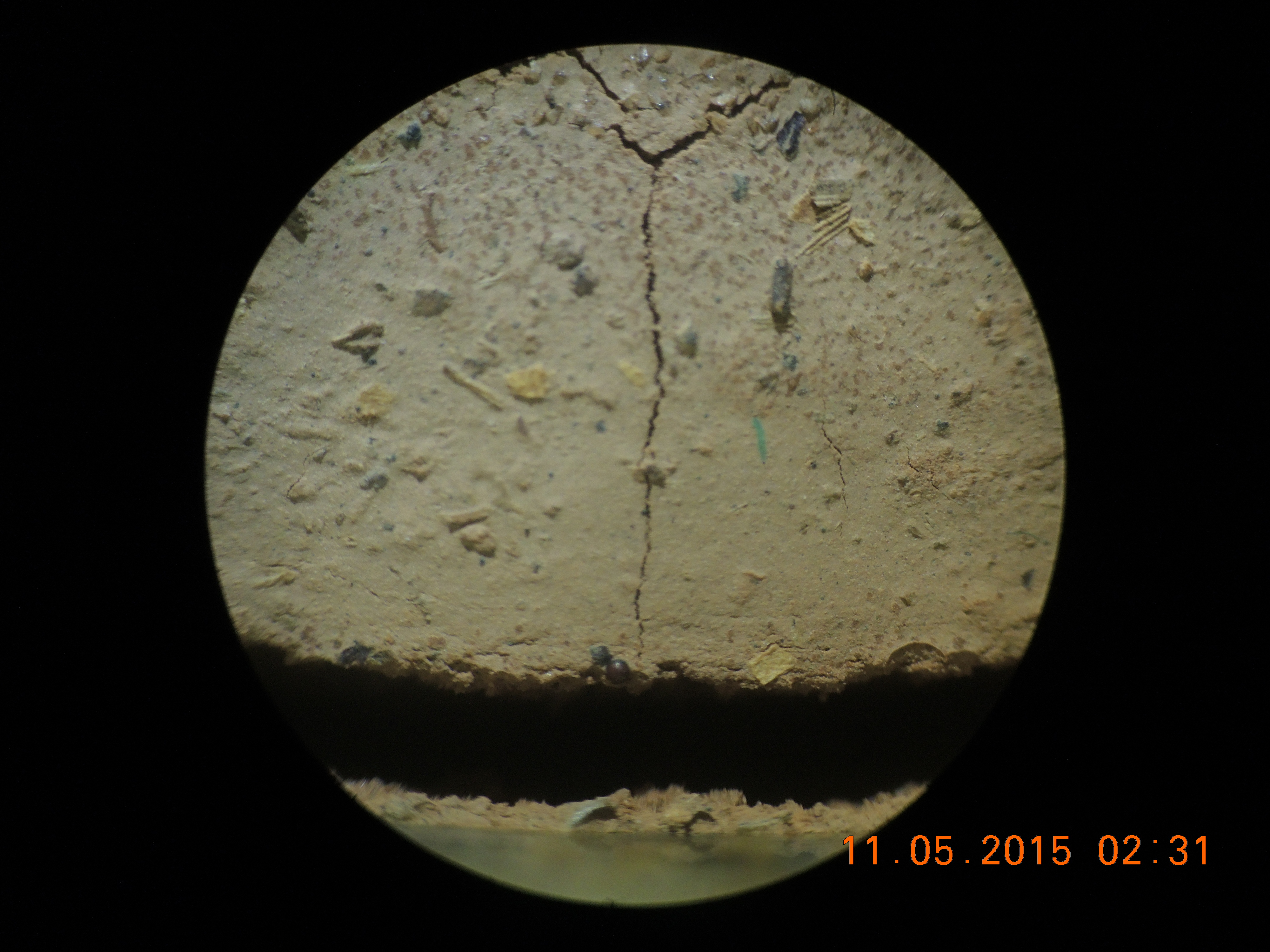

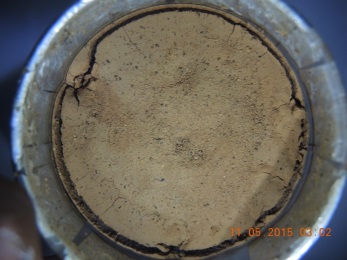

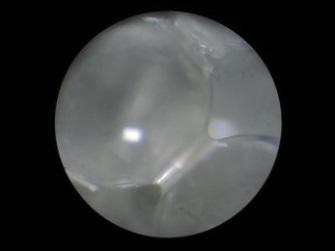


(a)

(b)

(c)

Figure S7 A single water bridge trapped (a) between two 1.40-1.50 mm diameter glass beads. The crust in this experiment was very weak. Top view of the porous medium (b) consisting of the natural sand. The zoomed view (c) shows the gap between the sand and the inner wall of the glass beaker.


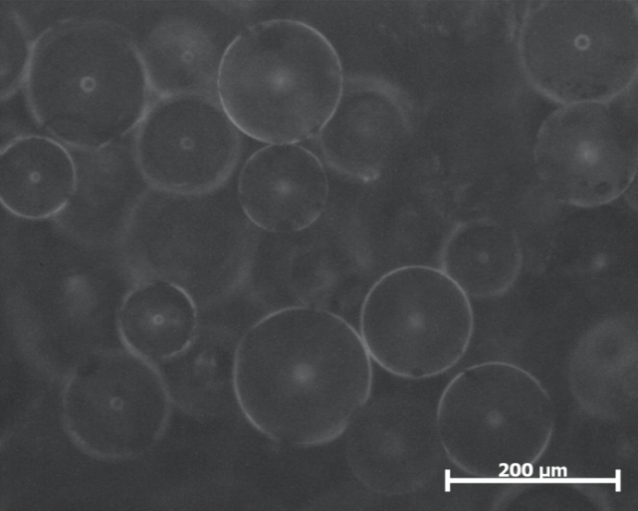


(a) With water

(b) With acetone


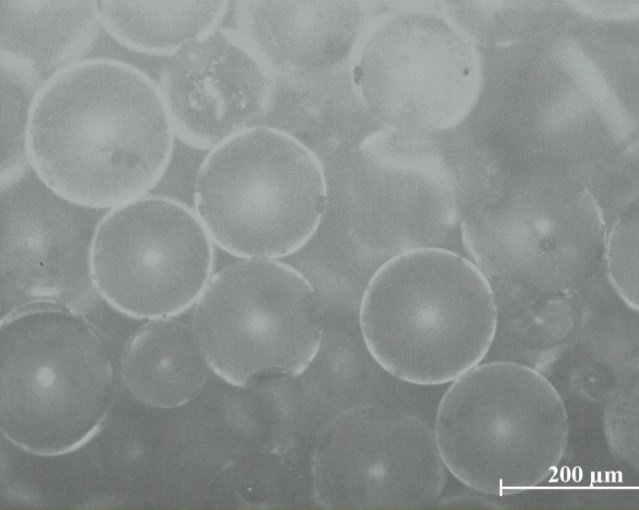


Figure S8 Microscopic images (1:1 ratio), with 100x magnification, of the top surface of thin upper crust after the end of the experiment. The porous medium consisted of 0.13 mm diameter GB. Evaporating liquid used was water (a) and acetone (b). Liquid trapped between the glass beads is hardly seen in either of the cases.


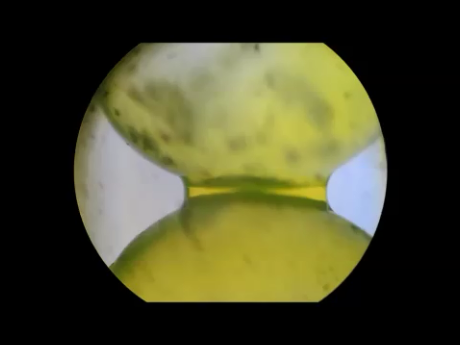

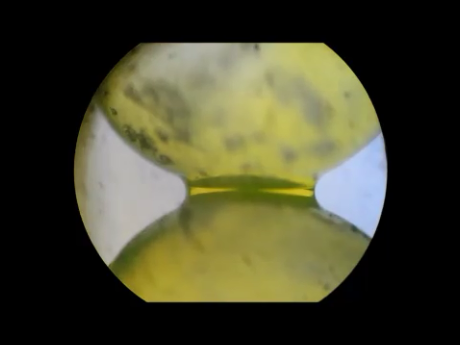


0 s

285 s


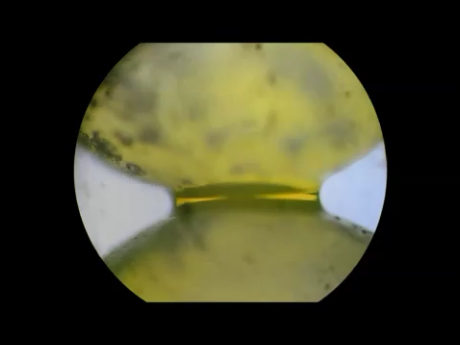


500 s


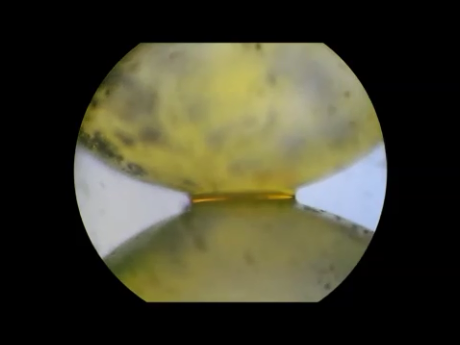


861 s


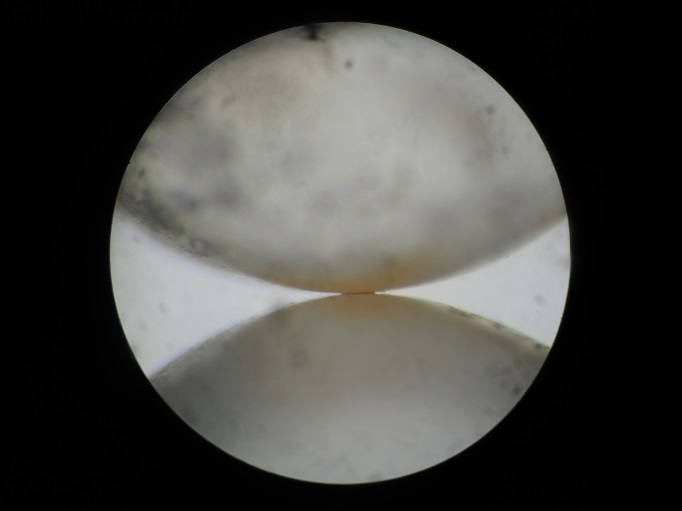


14 h


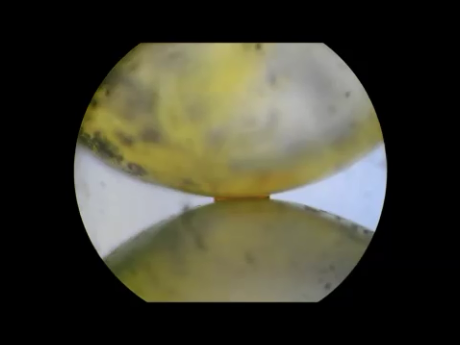


1069 s

A

B

C

D

E

F

Figure S9 Snapshots, taken at different times, of drying of a single water bridge trapped between two 3 mm diameter glass beads in contact. (a) and (b) were taken at 6x magnification while (c) – (f) were taken at 10x magnification. Water was coloured using fluorescein particles.


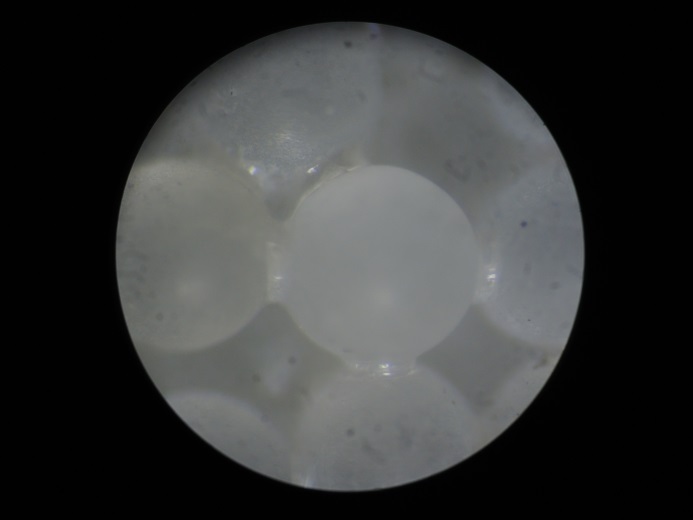

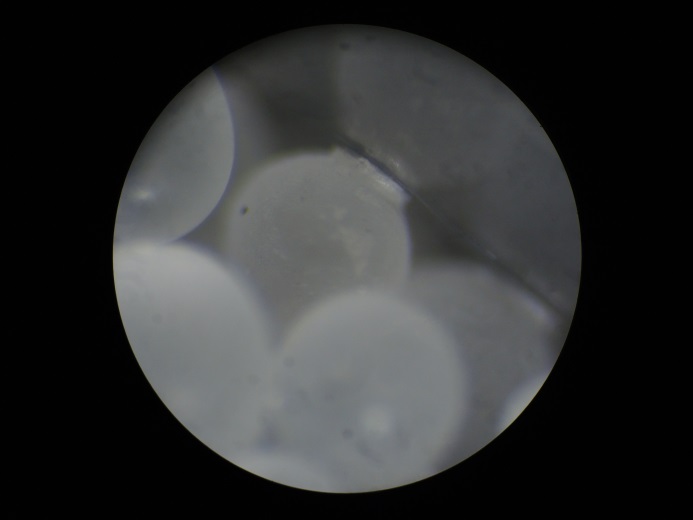


(a)

(b)

Figure S10 Microscopic images of the top surface of the crust formed near the upper exposed surface of the porous medium, taken after the end of the experiment. The porous medium containing hydrophobic 0.78 mm diameter glass spheres and water was heated from top. (a) shows the image with 10x, trapped water between two hydrophobic glass spheres are seen, and (b) shows an image near the wall with 10x, trapped water between the hydrophobic glass sphere and the hydrophobic glass wall can also be clearly seen.

R

$$\theta$$

r_1_

r_2_

R is the radius of the sphere while r_1_ and r_2_ are the radii of curvature of the liquid bridge in two principal directions.

h

Figure S11 The geometrical parameters of the liquid bridge trapped between two equal spheres of radius ‘R’. Also mentioned are the relations for the two principal radii of curvature of the liquid bridge.

**Supplementary Discussion:**

**Time of hardening – Bouncing stainless steel ball method**

The first question is *when crusts form and how to determine whether they are hard or not*? In the main file, we have shown that the hard layer starts forming as soon as the porous medium begins drying. For the other question, viz., to ascertain how hard the crust is, an experiment was conducted with water and 0.13 mm diameter hydrophilic glass beads in a small glass container. A higher density stainless steel (SS) ball, 1 mm in diameter, was dropped, at different instants, from a height of ~10 cm. Initially (when the porous medium was fully saturated) the ball didn’t bounce and made an indent (Figure S3) where it landed; this essentially signifies the softness at the beginning of the experiment. After some time, on the relatively drier regions (similar to the regions outside the white line in the IR images in Figure 2), the ball bounced back (Video S1 and S2) indicating the now hardened top surface; the porous medium was evaporating in stage 1 at this instant. This clearly indicates that the crusted layer formed at the top well before the experiment finished; this conclusion is in line with the explanation of Figure 2. Balls bouncing on the crusted top surface are seen in a much better way with multiple balls (Video S3).

## Evaporation characteristic curves for different porous media

Figure S4-S6 show the variations of different evaporation (of DI water) characteristic curves with time. Note that except the ‘sand’ experiment, all the other porous media were initially 100% saturated with water; for the ‘sand’ case about 12.50 g of excess water was put above the exposed sand surface. Evaporated mass curves are seen in Figure S4. The curves are typical of drying where a porous medium evaporates at a higher rate (Figure 1) till a certain instant after which the drying rate falls drastically to much lower rates. Higher drying rate regime is known as stage 1 while stage 2 is associated to the lower rates; transition regime connects these two periods. Note that in all the cases a gradual reduction in the evaporation rate is seen in stage 1 which has been recently shown [25] to be linked to the ‘shrinking evaporating patches’ patterns on the porous medium top surface. Figure S5 shows the variations of the near-surface (2 mm away from the top exposed surface within the porous medium) temperatures with time. During stage 1, the temperature increases gradually and achieves a much higher value in stage 2. Using the energy budget [25], it was shown that the gradually reducing drying rate leads to higher demand in the convective and radiative heat transfer from the top exposed surface to the ambient; this is achieved by increasing the surface temperature of the porous medium. For clarity, the near-surface temperature curves (Figure S6) have been plotted till t = 60 hours. Detailed discussion related to the nature of these evaporation characteristics curves are found in [25].

## Samples with 1.40-1.60 mm diameter glass beads and natural sand

Figure S7a is a microscopic view of a single water bridge trapped between two 1.40-1.60 mm diameter glass beads. The crust in this case, however, was very weak compared to the lower glass bead sizes. We believe that this weakness in the crust strength is due to the larger particle sizes. In the experiment with even larger spheres, 2.50-3.00 mm diameter glass beads, the crust did not even form. Figure S7b,c show images for the experiment with the natural sand. Unlike the glass beads cases (Figure 3a,b), the sand particles leave the glass inner wall during drying which led to shrinkage. This preferential motion of the sand particles appear to be similar to the ‘mudcracks’ (Figure S7b,c) found in the field during its desiccation. A circumferential uniform crevice (primary mudcracks) is seen in Figure S7b. Figure S7c shows the presence of the secondary mudcracks propagating orthogonally to the primary mudcrack. Interestingly, the crust with natural sand was not limited to a few layers near the top (as is the case with glass beads) but it covered the entire column (Figure 3d). In other words, the whole sample was fully crusted. The crust was surprisingly much harder compared to the hardest crust in case of the glass beads (0.13 mm diameter). The irregular particle structure and their higher mean level of roughness values should have resulted in the frictional locking throughout the sample. This (mechanical locking) must have increased the strength of the crust in case of the natural sand.

## Absence of external heating (0.13 mm GB)

Two experiments of evaporation, in the ambient with no external heating, were conducted with 0.13 mm diameter GB, each with DI water and acetone as the evaporating liquid in a 5 ml vial. Glass beads contained nearly 70% SiO_2_ which forms a weak layer of SiOH when exposed to the ambient. The –OH group thus can interact with –H group of water and form hydrogen bonds which could be one of the major reasons behind the adhesive strength of crust. The evaporating liquid was acetone, a ketone group member, which have neither –H nor –OH group. In both the cases, however, we observed the presence of a weakly crusted upper layer. Figure S8 shows the optical microscopic images, 100x magnification, of the top surface of the crusted samples. Trapped water between the glass spheres can hardly be seen in these images either with water (Figure S8a) or with acetone (Figure S8b). Note that the crust strength with acetone was much weaker than with water case, which was much weaker than the crust formed with water in the presence of external heating.

**Drying of a single water bridge**

Figure S9 shows time-sequence snapshots of drying of a water bridge. The water bridge is trapped between two 3 mm diameter glass beads in contact. The bridge appears green because of the fluorescein particles; these particles are red in colour but turn water green. The snapshots were captured under a microscope with the bottom light switched on; this light provided external heat for the drying. Figure S9a is the initial condition i.e. t = 0 of the drying process. The water bridge is seen clearly and is in pendular form i.e. the bridge is not in contact with the flat glass plate placed below the glass beads. Figure S9b shows the bridge condition after ~5 minutes. Two significant developments can be seen here: (1) radius of curvature of the left and right menisci has reduced considerably compared to that in Figure S9a and (2) the length (horizontal span) of the bridge has reduced due to drying. We can define this length as the ‘bridge radius’; if we assume the bridge to be a disc. At this instant the microscope magnification was increased (from 6x to 10x) so that the dimensions of the (reduced) water bridge could be captured properly. Furthur reduction on the bridge radius can be seen in Figure S9c (t ~ 8 minutes) and Figure S9d (t ~ 14 minutes). At around 18 minutes (Figure S9e) the water bridge is too small. Interestingly, the bridge did not evaporate completely. Figure S9f shows the snapshot, captured after ~ 14 hours, where a tiny amount of water is unevaporated; in fact this water cannot be evaporated further in similar conditions. Note that if the spheres are separated by a finite distance (at least till the order of beads size) then the unevaporated water cannot be found. This unevaporated water provides strength to the system and in a system with much higher number of beads this become significant; like a crust in the present experiments (see main article).

**Effect of hydrophobicity in determining the crust strength**

Figure S10a shows a central HO glass sphere connected to four similar spheres via the unevaporated liquid bridge. Figure S10b shows a HO glass sphere connected to the HO glass beaker wall through the trapped water between them. This type of connection was found throughout the porous medium.

A hydrophobic system was supposed to repel water and not trap any water content between the HO glass spheres. However it not only trapped water between the HO spheres but the crust formed as a result was much stronger than that of a hydrophilic (HI) system made out of similar HI glass sphere sizes. One of the probable reasons could be the strength of the hydrophobic-hydrophobic (HO-HO) interactions which are stronger than both the hydrogen bonding and the hydrophobic-hydrophilic (HO-HI) interaction. Since during evaporation, water content keeps on reducing with time exposing the individual HO glass sphere. Two nearby HO glass spheres which have been exposed (partially surrounded by water) start interacting with each other using the HO-HO interaction. In the process they might stick to each other which possibly lead to water entrapment between the HO glass spheres. Since the HO-HO interaction is stronger, the water trapped between the HO glass spheres finds it difficult to evaporate as compared to the case in which water is trapped between the HI glass spheres. The HO-HO interaction might also have suppressed the molecular motion within the trapped water content.

One of the most surprising results was the formation of the crust in a porous medium containing hydrophobic spheres. In fact for the same glass spheres sizes (like 0.78 mm diameter), the crust strength in case of hydrophobic glass spheres was much stronger than the hydrophilic counterpart. We justified it by using the fact that HO-HO interaction is much stronger than the HO-HI interaction in which case water still gets trapped between a pair of hydrophobic spheres. The higher HO-HO interaction further enhances the crust strength by not letting trapped water either to move or evaporate.

**Mass of water trapped in the crust**

The liquid bridge is seen in Figure S11. We can estimate the bridge volume, by assuming it to be a disc, as:

$$V_{LB}=\pi r_{2}^{2} h=2\pi R^{3}{sin\theta tan}^{2} \theta{\tan\left( \frac{\theta}{2} \right)\left( 1-\tan\left( \frac{\theta}{2} \right) \right)}^{2}$$

We estimated $r_{2}\sim27\mu m$ (on an average) based on the microscopic images for the case with$R\cong65\mu m$. Using these values we calculated$\theta\sim{29.4}^{0}$. The volume of the trapped water bridge is$V_{LB}\sim3.84*{10}^{-14}$ m^3^ and its mass is$m_{LB}\cong3.84*{10}^{-11}$ g. The corresponding volume and mass of one glass ball is$V_{GB}\cong1.37*{10}^{-12}$ m^3^ and$m_{GB}\cong3.43*{10}^{-9}$ g respectively. The weight ratio$m_{LB}/m_{GB}$ is therefore$\sim1.1\%$. This weight ratio was also determined using the thermogravimetric analysis (see the main article file). The weight of a single glass bead (0.13 mm diameter) is $W_{GB}\cong3.77*{10}^{-13}$ N.

**Force in the crust based on the microscopic images**

We now estimate the order of magnitude of different types of forces involved in keeping the crust intact. These forces may be due to: (A) molecular attraction or repulsion (van der Waals force), (B) surface tension, (C) charges (electrostatic force), (D) roughness (mechanical locking), and (E) hydrogen bonding. We give estimates of these forces based on the images captured using SEM. We also estimate the (average) weight (and volume) percentage of water in the water bridge.

1. **Van der Waals force**

Magnitude of the van der Waals ($F_{VW}[N]$) force between two spheres of equal radii is given as,

$$F_{VW}=\left( \frac{AR}{12d^{2}} \right)$$

Where, A (typically of the order of${10}^{-20}$J) is the Hamaker constant and d [m] is the separation between the two spheres. For the spheres (having 0.1 mm diameter) in contact we can assume$d\sim1nm$ which would give$F_{VW}\sim O\left( {10}^{-6} \right)N$.

1. **Force due to surface tension**

The expression for the pressure inside the meniscus (Figure S11) produced by surface tension ($\sigma$) is,

$$P=\sigma\left( \frac{1}{r_{1}}-\frac{1}{r_{2}} \right)$$

Where, r_1_ and r_2_ can be written as:

$$r_{1}=R\left[ \frac{1-cos\theta}{cos\theta} \right]=R tan\theta\tan\left( \frac{\theta}{2} \right)$$

$$r_{2}=R tan\theta-r_{1}=R tan\theta\left[ 1-\tan\left( \frac{\theta}{2} \right) \right]$$

Figure S11 describes the details of R and$\theta$. We can write the total pull between the two spheres as,

$$F_{ST}=P*\pi r_{2}^{2}=2\pi R\sigma\left( \frac{1-2\tan\left( \frac{\theta}{2} \right)}{1+\tan\left( \frac{\theta}{2} \right)} \right)$$

Using SEM images for the water bridge, we get$F_{ST}\cong1.11*{10}^{-5}N$ for beads with 0.1 mm diameter.

1. **Electrostatic force**

It is difficult to calculate the electrostatic force between two spheres which contains impurities in unknown amount and types. Further, with a liquid bridge in between the spheres, estimating this force becomes harder. However, for the clean experiment (where clean glass beads were used in a clean vessel with millipore water as the evaporating liquid) the charge can only come from the ambient. Based on the rough estimates of charge in the air, we get the magnitude of the electrostatic force:$F_{ES}\sim O\left( {10}^{-6} \right)N$ or even lower.

1. **Force due to mechanical locking**

Due to the presence of the liquid bridges in the contacts of the spheres, the spheres would not be mechanically locked. However, with such large numbers of spheres (like the ones shown in the main file), the spheres will be constrained and thus loose the degree of freedom. We can say that the crust would be mechanically held rather than mechanically locked.

1. **Force due to hydrogen bonding**

We estimate the energy, rather than force, of attraction due to the hydrogen bonding. The hydrogen bond enthalpy is 23.3 kJ/mol i.e. ~1.3 kJ/g. Hydrogen bond enthalpy is therefore$U_{HB}\cong4.99*{10}^{-8}$J. For comparison, van der Waals energy is$U_{VW}\cong5.41*{10}^{-16}$J. The hydrogen bond is therefore much stronger than the van der Waals forces.

**Surface energy of different systems**

1. **Surface energy of glass beads**

The surface energy of a single glass bead (0.13 mm diameter) can be estimate as:

$$U_{GB}=310*4\pi R^{2}\cong1.65*{10}^{-8}J$$

Where, the surface energy of the glass has been used to be 310mJ/m^2^.

1. **Surface energy of water**

The surface energy of water can be estimate as:

$$U_{GB}=\sigma*2\pi\left( r_{2}^{2}+r_{1}^{2} \right)\cong3.72*{10}^{-10}J$$

**Reason for the formation of crust**

From the above arguments, we note that the surface tension force is at least an order of magnitude higher than the van der Waals and electrostatic forces. However, in case of water, the energy stored in the form of the hydrogen bonds dominates since it is at least two orders of magnitude higher than the surface energy of the trapped water. We therefore conclude that the strength of the crust is due to the surface tension and hydrogen bonding of water. The magnitude of all the forces is much higher than the weight of a single glass bead. Note that for liquids such as acetone, the hydrogen bonding enthalpy would be zero while the surface tension would decrease significantly compared to water. In this case the crust would be very weak as observed in the experiments.

We still do not know why we get an unevaporated liquid bridge. A kinetic model of the energy might answer this question.
