# Supplementary material for: Formation of a hard surface layer during drying of a heated porous media
Source: PLoS One. 2020 Feb 27;15(2):e0229723. doi: 10.1371/journal.pone.0229723 (PMC7046278; doi:10.1371/journal.pone.0229723)
Supplement: Attachment — Submitted filename: Response Referee 2.pdf [file pone.0229723.s007.pdf]

Response to #Reviewer 2

First of all we would like to thank the Referee for raising a few concerns while reviewing our manuscript. These concerns helped the manuscript in achieving a better clarity. Majority of the concerns have now been addressed in the manuscript. Our comments to the points raised by the Referee are given below.

COMMENT 1: As this paper concludes that the liquid bridge, or the capillary bridge, is the dominant contributor to the surface hardening, then the surface water wettability should be a major factor in the hardening result. Authors are using 'hydrophobic' spheres for most of their experiments.

(a) Could the authors provide some characterizations, such as contact angle, of the surface wettability before and after the hydrophobic treatment? The hydrophobic surface should have a water contact angle higher than 90 degrees. Maybe after the treatment, even though the contact angle increases, it is still less than 90 degrees and cannot be considered a 'hydrophobic' surface.

(b) Also, In Figure S11, the authors discuss a scheme for hydrophilic surface but not a hydrophobic surface.

RESPONSE: We have used 'hydrophilic' particles in most of the experiments. We only used 'hydrophobic' spheres in one experiment. The observations (that, surprisingly, this crust was harder in this case as compared to its hydrophilic counterpart) of this experiment were mentioned in the supplementary information file.

As of now, we have no understanding of this type of behaviour. The initial thought behind using the hydrophobic particles was to (possibly) avoid the formation of the liquid bridges. However, the bridges did form. This work has in fact opened a very interesting research field i.e. the formation of liquid bridge between hydrophobic spheres.

(a) Unfortunately, we did not measure the contact angles as this is a dynamic property. With evaporation, we observed the change in the curvature of the liquid meniscus; this was observed in the experiment where evaporation from a (coloured) liquid bridge was studied between two hydrophilic 3 mm diameter glass beads. Please see Figure S9 and Video S4. The microphysics of the (dynamic) contact angle in this case is rather complicated but is expected to remain similar to the physics of evaporation from a sessile droplet where contact angle depends on the local roughness levels.

To give an idea of the contact angle, an accepted value of 20^0 can be safely assumed for the hydrophilic glass beads.

Also, since our interest was in studying the effect of hydrophobicity in large samples, we never tried measuring the contact angle in this case; we just trusted the well-accepted method to make the glass beads hydrophobic. We would like to add that the beads were indeed hydrophobic because a cluster of these beads were able to trap some air (in the form of

bubble) with it when dropped water. We had to use an ultrasonicator to remove these air bubbles.

(b) We are, at the moment, trying to work out the physics of the formation of a liquid bridge between two hydrophobic spheres. The forces can then be figured out.

We also want to highlight that we are trying to understand the physics of evaporation from a liquid bridge (formed between two hydrophilic / hydrophobic spheres, or a combination of the two, in proximity) and are planning to communicate a separate article.

COMMENT 2: This reviewer assumes that comparing to strength; the hardness may be a more important parameter describing the hardening process. In page 6, section-time of hardening-bouncing stainless steel ball method, the authors use the dropping ball methods to describe the hardening process, do authors have enlarged surface image after the ball 'indentation'? Have authors tried any standard hardness measurement to characterize or these indentation images to calculate the hardness?

RESPONSE: We did try to test the hardness of the crust using general purpose indenter devices. We used 1 mm steel balls to test the crust strength in 0.13 mm glass beads case. Note that the indenter (steel ball) is ~7.5 times bigger than the glass particles.

Due to the random distribution of the three phases (solid glass, liquid in the liquid bridge, and air), we found it very difficult to carry out the hardness measurements. Currently, we are trying to come up with a more reliable technique to measure the hardness. We hope to communicate the hardness values in near future.

Please note that the steel ball dropping experiment did not make any indent on the dry surface. It, however, did indent the completely wet surface, which was expected.

COMMENT 3: On page 7, first sentence, 'these clumps could not be held between the fingers as they fell apart' is not a very scientific expression, please rephrase.

RESPONSE: We just wanted to state that the clump had very little strength; the finger method might have given some idea to the readers about its strength. However, as suggested by the referee, we have rephrased this sentence. This sentence now reads – "Obviously, unlike the crusts, these clumps fell apart easily".

COMMENT 4: Figure 3, please provide high-resolution images and clearly label the images, such as marking important features in the image, add scale bar, etc. Right now it's very unreadable.

RESPONSE: Thank you for this suggestion. Please see the updated Figure 3.

COMMENT 5: Figure 4b, please add scale bar, Figure 4c, d, please enlarge the scale bar.

RESPONSE: Thank you for the suggestion. Please see the updated Figure 4.

COMMENT 6: Figure 5b, is the centre brown region the crusted thin upper layer? If so, please mark carefully.

RESPONSE: This figure is now Figure 7. As suggested by the referee, we have marked the key regions. Please see this updated figure.

COMMENT 7: Figure 6, again, please label clearly on all subfigures, and please provide a scale bar for d and e.

RESPONSE: This figure is now Figure 8. As suggested by the referee, we have included these details. Please see the updated Figure 8.
